# Visual body size estimation in adolescent anorexia nervosa: Behavioural and neurophysiological data suggest intact visual perception and biased emotional attention

**DOI:** 10.1038/s41398-024-03144-y

**Published:** 2024-10-18

**Authors:** Hugo Romero Frausto, Isabel Rahder, Anke W. Dalhoff, Kati Roesmann, Georg Romer, Markus Junghöfer, Ida Wessing

**Affiliations:** 1 Department of Child and Adolescent Psychiatry, University Hospital Muenster, Schmeddingstraße 50, 48149 Muenster, Germany; 2 Institute for Clinical Psychology and Psychotherapy, University of Siegen, Obergraben 23, 57072 Siegen, Germany; 3 Otto Creutzfeldt Center for Cognitive and Behavioral Neuroscience, University of Muenster, Fliednerstr. 21, 48149 Muenster, Germany; 4 Institute for Biomagnetism and Biosignalanalysis, University Hospital Münster, Malmedyweg 15, 48149 Münster, Germany

**Keywords:** Psychiatric disorders, Scientific community

## Abstract

Body image disturbance is a key symptom of anorexia nervosa (AN). AN patients report body dissatisfaction and overestimate their own body size in several tasks. This study aimed to clarify whether this overestimation arises from deficits in visual perception. To this end, 36 adolescent restrictive-type AN patients and 42 matched healthy controls performed metric and depictive body size estimation (BSE) tasks. Magneto- and electroencephalography were measured during the size estimation of 66 computer-generated body pictures varying in size from underweight to overweight. AN patients versus controls showed overestimation across self-referential metric and depictive BSE tasks, but similar performance in a depictive BSE task without self-reference and similar early neurophysiological responses. Starting mid-latency (200 ms), AN patients showed relatively more neural activity in response to underweight body pictures and less neural activity in response to higher-weight body pictures in distributed brain regions. A secondary comparison of AN patients with slight vs. distinct overestimation during self-referential BSE uncovered relatively stronger neural responses to body pictures corresponding to the estimated body mass index. These results suggest that body image disturbances in adolescent restrictive-type AN patients depend on self-reference and do not represent a deficit of visual perception, but rather biased emotional attention.

## Introduction

Anorexia nervosa (AN), an eating disorder usually beginning in adolescence, is characterised by significantly low body weight, food intake restriction, and disturbances in how one’s body shape is perceived. Body image disturbance (BID) is an important risk factor [1] and predicts the onset of an eating disorder [2]. Moreover, BID persistence after successful AN treatment predicts relapse [3] and long-term outcomes [4, 5]. However, despite its high clinical relevance, the aetiology and nature of BID are still poorly understood.

Researchers have long discussed whether BID in AN is mainly caused by disturbances in the cognitive-affective domain, e.g., overly strong slender ideals and body dissatisfaction, or whether perceptual disturbances are at play [6]. Regarding perceptual disturbances, many studies have shown that AN patients overestimate their own body size in tasks of body size estimation (BSE), in which a body picture representing one’s own body must be selected from a set of body pictures of varying sizes [7]. This has led to the assumption that AN patients might have impaired visual perception in general or of bodies in particular [8]. However, visual perception involves a complex interplay of bottom-up (perceptive) and top-down (cognitive) processes that are difficult to disentangle [6].

The role of visual body processing in AN might be better understood by elucidating the underlying neural mechanisms. Functional magnetic resonance imaging (fMRI), for example, allows experimental effects to be assigned to regions known to be involved in visual perception vs. cognition and emotion. In healthy participants, fMRI has identified a specialised neural network for visual body processing [9], including body-selective regions in the ventral temporal cortex: the extrastriate body area (EBA) and the fusiform body area (FBA). While the EBA is more sensitive to body parts and the FBA to whole bodies, both regions involve the visual processing of body posture and shapes. More elaborate cognitive-affective processes have also been ascribed to these regions, but this evidence is less clear [9, 10]. It seems likely that such processes rely on functional integration within an extended neural network, including prefrontal, parietal, and temporal regions associated with executive control and social cognition [9].

So far, several fMRI studies have used body stimuli to investigate BID in AN, but the results vary. Indeed, several fMRI studies have reported reduced neural activity in visual occipito-temporal regions, including EBA and/or FBA, in AN in response to non-self-body pictures [11–14] or to pictures of one’s own body in particular [15, 16]. Such results led two reviews to conclude that disturbances of the neural network for visual body processing might influence BID in AN [17, 18]. Yet, many other fMRI studies using body stimuli have not found any disturbances of this visual network but instead have reported alterations in neural networks associated with cognition (e.g., PFC) and emotion (e.g., amygdala, insula, ventral striatum [19–25]). This heterogeneity might be explained by divergent experimental designs, small sample sizes, different tasks, and different stimuli. Nearly all fMRI studies investigated adults or mixed adolescent and adult samples, and only one study considered possible specifics of adolescents [23]. Many studies focussed explicitly on cognitive-affective aspects were not designed to elucidate visual body processing per se.

In addition to hemodynamic imaging, electro- (EEG) and magnetoencephalography (MEG) can be used to elucidate the neural mechanisms of visual body processing in AN. Their high temporal resolution supports differentiating earlier bottom-up (perceptive) processes from later top-down (cognitive) processes in time and in space via source localisation. However, studies of BID in AN using EEG or MEG are rare. Again, studies have used different methods, and most studies have focussed on late event-related potentials (ERPs) like the late positive potential (LPP, >300 ms), thus targeting the cognitive-affective aspects of BID. Regarding visual perception in general, two studies used face and house stimuli [26, 27] and reported reduced early (P100, <150 ms) visual ERP amplitudes in adult AN patients. Underlying neural sources were localised in the visual cortex, including the fusiform cortex near the EBA, suggesting deficits of early visual perception in AN [27]. However, an ERP study in adolescent AN patients presented schematic rabbits and trucks in a card-sorting paradigm and revealed that P100 responses were increased in AN [28]. Overall, these results await replication and do not necessarily apply to body stimuli. Regarding cognitive-affective BID, two studies used shape-/weight-related words [29] and body pictures [30] and found no AN-specific ERP components. Another two studies [31, 32] presented body pictures in different weight categories and observed that adolescent and adult AN patients, unlike HC, showed the highest LPP amplitudes for underweight pictures, followed by normal-weight and overweight body pictures. This was attributed to a greater capture of attention by disorder-relevant stimuli in AN. Overall, of the few EEG studies related to BID in AN, two suggest a general disturbance of early visual processing, two found no effects, and two suggest a later attention bias to underweight bodies, presumably driven by cognitive-affective processes. To date, not enough EEG/MEG studies have focussed on the neural correlates of visual body perception and BSE in AN.

The present study aimed to fill this gap by investigating estimated neural source activity based on parallel EEG and MEG^1^ recordings during BSE in adolescent AN patients and age-, gender-, and IQ-matched controls. We utilised standardised computer-generated body pictures of varying body mass indices (BMIs) [34] in a viewing task, a self-referential depictive BSE task, and a non-self-referential depictive BSE task. To ensure group comparisons were unaffected by deviating stimulus properties or procedures, participants viewed the exact same stimuli, and BSE tasks only differed in their instructions. Clinical characterisations were complemented by self-reports on BID and a metric BSE task. We expected AN patients to show higher BID in self-reports and self-referential BSE tasks. If visual body processing per se is affected, AN patients will show stronger discrepancies in the non-self-referential depictive BSE task as well. Further, a convergent general disturbance of the neural network of visual body processing will be indicated by reduced neural activity at earlier components (starting ~100 ms) and in occipito-temporal regions across tasks. Conversely, disturbances of cognitive-affective aspects of body image will predominately be reflected in later components (starting ~300 ms) and in an extended neural network, including prefrontal and posterior parietal regions. Moreover, neural correlates of cognitive-affective BID will manifest particularly in the self-referential depictive BSE task.

## Methods

### Participants

AN patients were recruited during inpatient treatment on a specialised ward for eating disorders at the Department of Child and Adolescent Psychiatry, University Hospital Muenster, Germany. Diagnoses were confirmed using structured clinical interviews. Patients with comorbid conditions were included, except for pervasive developmental disorders or psychotic disorders. Age-, gender-, and IQ-matched healthy controls (HC) were recruited from secondary schools in Muenster. Exclusion criteria for HC were clinically relevant psychopathology (telephone screening, structured clinical interviews, questionnaires) and over- or underweight (BMI age percentile >90 or <10). General exclusion criteria were intellectual disability, suicidality, substance abuse, and MEG/EEG-related exclusion criteria^2^. All participants and their parents were informed of the study protocol and gave written informed consent. The study was approved by the ethics committee of the Medical Association of Westphalia-Lippe, in accordance with the Declaration of Helsinki (2016-457-f-S).

### Clinical characterisation

#### Structured clinical interviews

Diagnoses were confirmed using the Eating Disorder Examination Interview (EDE-I [35]) and the Diagnostic Interview for Mental Disorders in Children and Adolescents (Kinder-DIPS [36]).

#### Questionnaires

Participants completed German versions of the Eating Disorder Inventory for Children (EDI-C [37]), the Body Shape Questionnaire (BSQ [38]), Beck’s Depression Inventory (BDI-II [39]) and the Screen for Child Anxiety-Related Emotional Disorders (SCARED-D [40]).

#### Intelligence test

Participants completed a computerised German version of the Cattell’s Fluid Intelligence Test, Scale 2 (CFT-20-R [41]).

#### Metric body size estimation task

In a metric BES task [42], participants had to estimate the circumference of their own waist, upper arm, and thigh, employing a rope to make a circle. After that, the real circumference of each body part was determined.

### MEG/EEG experiment

#### Procedure

**Fig. 1:**
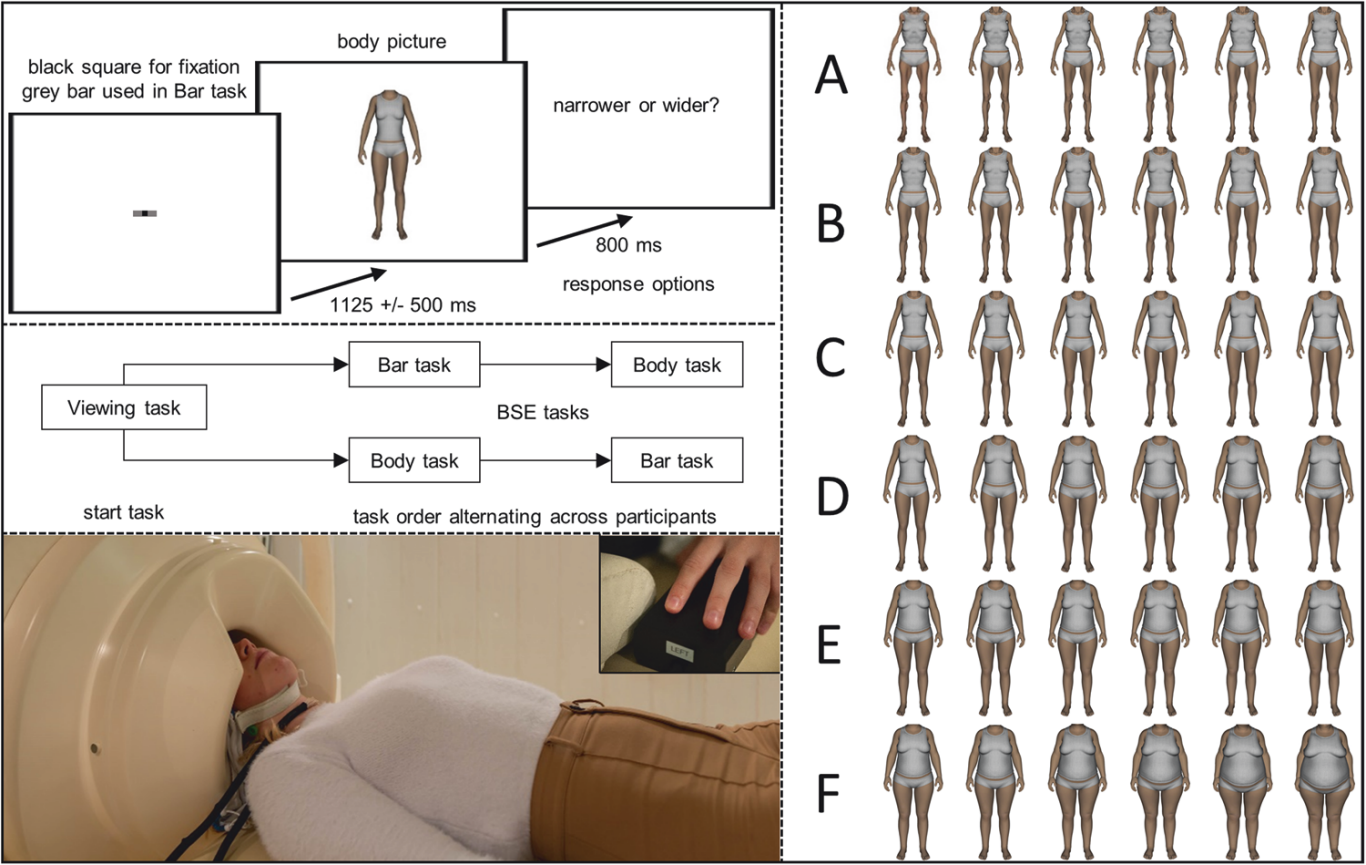
Experimental paradigm. Top left: Procedure. Participants were asked to attentively view the body pictures. In the viewing task, no further instruction was given, and no response options were presented. In the BSE tasks, participants used the response options “narrower” or “wider” and responded via button press. In the bar task, participants compared the width of the waistline of the previously presented body picture to the width of the central grey bar. In the body task, participants compared the previously presented body picture to their own body. Presentation order of the BSE tasks alternated across participants. Bottom left: Subject during parallel EEG and MEG recording and response device. Right: Body pictures in categories A–F from top to bottom.

Participants were introduced to the MEG chamber and tasks. Digital head renderings were recorded using a 3D tracking device Participants wore an electrode skullcap for parallel EEG measurements. Participants were placed in supine position in the MEG scanner with their nasion 86 cm from a TFT screen (Fig. 1).

#### Stimuli

Sixty-six pictures were selected from the body-only computer-generated pictures of women database, which contains pictures with normalised visual properties (brightness, complexity, contrast) and BMI information for the depicted bodies [34]. Body pictures were classified into six categories: A (BMI 12.19–14.74), B (BMI 14.77–17.17), C (BMI 17.19–19.78), D (BMI 19.80–29.91), E (BMI 29.92–32.45), and F (BMI 32.46–56.26).

#### Experimental paradigm and depictive body size estimation tasks

The experiment always started with a viewing task followed by two depictive BSE tasks, namely the self-referential body task and the non-self-referential bar task. The BSE task order alternated across participants. In all tasks, 66 body pictures were presented for 800 ms each with a jittered inter-stimulus interval (1250 ± 500 ms) in a pseudo-randomised order (maximum three consecutive stimuli per category, equal transition probability). Each stimulus was repeated three times, resulting in 198 total trials per task. Pictures were presented with a vertical visual angle of 9° while participants kept their eyes focused on a central black square inside a grey bar. This bar was used as a comparison in the bar task and had the same width as the waist of the body picture representing a median BMI of 19.79 (N000 [34]).

During all tasks, participants attentively viewed the pictures. In the viewing task, no further instruction was given. In the BSE tasks, participants responded via button press with the response options “narrower” or “wider”. The assignment of response options to the middle or index finger of the left hand alternated across participants. In the bar task, participants compared the size of the previously seen body picture (at the waist) to the size of the central grey bar. In the body task, participants compared the size of the body picture to their own body size. All stimulus presentations and tasks were performed using Presentation software version 14.8

#### MEG/EEG data acquisition and analysis

Visually evoked magnetic fields (VEMF) were recorded using a 275-channel whole-head sensor system MEG with first-order axial SQUID gradiometers (Omega 275, CTF MEGTM, VSM Medtech Ltd., Coquitlam, British Columbia, Canada). In parallel, visually evoked potentials (VEP) were recorded using either an 80-channel (19 HC, 20 AN) or, after technical modifications of the MEG/EEG system, a 57-channel (27 HC, 18 AN) EEG skullcap (Easycap GmbH, Germany), according to the International 10–20 system ([43], supplement S1). During EEG recording, FCz was used as an online reference point and re-referenced offline to an average reference. Head position and head movements were tracked using landmark coils in each ear channel and on the nasion. Continuous MEG/EEG data were recorded from 0 to 150 Hz using a sample rate of 600 Hz and then downsampled to 300 Hz. A zero-phase Butterworth third-order high-pass filter [12 dB/oct] and a fourth-order low-pass filter [24 dB/oct]) with cut-off frequencies of 0.1 and 48 Hz, respectively, were applied. A fourth-order bandstop filter with a frequency of 60 Hz and a width of 3 Hz was applied offline to suppress artefacts of the stimulus presenting monitor and its harmonics. Single-trial data editing and further artefact rejection were conducted using the method for statistical control of artefacts in high-density MEG/EEG data [44]. Single epochs of 800 ms (200 ms before to 600 ms after stimulus onset) were averaged corresponding to experimental conditions. A pre-stimulus interval of 150 ms was used for baseline adjustment (overview of EEG electrode and MEG sensor data: supplement S2).

Neural sources underlying the VEMFs/VEPs were estimated using the L2-Minimum-Norm approach (L2-MNE [45]) with a spherical shell consisting of 350 evenly distributed dipole pairs (MEG) or dipole triples (EEG) as the source model^3^. A source shell radius of 87% of the individually fitted head radius was chosen, which roughly corresponds to the grey matter depth. Leadfield matrices were calculated for all participants and conditions using a Tikhonov regularisation parameter lambda of 0.1 for MEG and 0.2 for EEG data. The estimated neural activity was calculated as the vector length of each dipole. Topographic maps displaying the direction-independent current dipole activity [46] were calculated for each participant, condition, and time point based on averaged magnetic/electric field distributions and individual sensor positions.

### Statistical analyses

#### Metric body size estimation task

The measured and estimated circumferences of each body part were used to calculate body perception indices (BPI = estimated/measured × 100 [47]), and their mean determined the total metric BPI. An independent samples *t*-test compared total metric BPIs between groups (AN, HC).

#### Depictive body size estimation task

In each depictive BSE task, the estimated body picture BMI was determined by fitting a Weibull cumulative distribution function *f*(*x*) = 1−e^(−(*ax*)*β*)^ ([48]; executed in MATLAB) to the dichotomous answers (“narrower”, “wider”) in 196 trials (66 body pictures × 3 repetitions). This resulted in a fitted BMI value for each participant and task that was used to calculate a BPI (body task BPI = fitted BMI/actual BMI × 100; bar task BPI = fitted BMI/19.61 [BMI of the picture matching the bar width]×100). The resulting BPIs were analysed using a repeated measures ANOVA with the factors *task* (body, bar) and *group* (AN, HC)^4^.

#### MEG/EEG data

Statistical MEG/EEG data analyses calculated repeated measures ANOVAs with the factors *body picture* (A–G), *task* (viewing, bar, body) and g*roup* (AN, HC) for each estimated source and time point. We controlled for multiple testing via a non-parametric cluster-permutation analysis as described in Maris and Oostenveld [49], with a significance criterion of *p* < 0.05 on both sensor- and cluster-level. Analyses concerned the whole brain and a time window from 50 to 550 ms, avoiding stimulus onset and offset effects resulting from low-pass filtering. For clarity, clusters adjacent in time and space and showing identical effects were merged. For visualisation, clusters were projected on a standard 3D brain model. Pre-processing and analysis of MEG/EEG data were done using the MATLAB-based software EMEGS version 3.1 (emegs.org [50]). L2-MNE data within each cluster were extracted for post hoc analyses.

#### Secondary analysis

To further investigate the effect of body size overestimation on a neural level, we performed a secondary analysis of an EEG cluster showing a group-specific effect (see results). Two AN subgroups were built via a median split on body task BPIs (median BPI = 124.98): Group AN1 showed slight overestimation, comparable to HC, and group AN2 showed distinct overestimation (sample characteristics: supplement S3). To focus on the effect of self-reference, only the two BSE tasks were included in an ANOVA with the factors *body picture* (A–G), *task* (bar, body) and *group* (AN1 and AN2).

#### General statistical considerations

The required sample size for this study was determined using G*Power 3 [51] based on effect sizes from Horndasch et al. [32] (details see S4). All statistical analyses, unless otherwise described, were calculated using SPSS 27 and used a significance criterion of *p* < 0.05. Post hoc tests included 2-sided *t*-tests and, for the factor BMI, planned polynomial contrasts testing for linear and quadratic trends. ANOVA results were Greenhouse–Geisser corrected if necessary. For *t-*tests, the assumption of normal distribution was not tested, as sample sizes were above *N* = 30 (except one group of *N* = 29), and *t-*tests are known to be robust in the face of violations of this assumption [52].

## Results

### Subjects’ characteristics

We recruited 40 AN patients and 59 HC participants. Of these, 4 AN patients and 17 HC participants were generally excluded. Moreover, depending on the kind of data, different numbers of participants were excluded due to insufficient data quality (supplement S5). To maximise statistical power, all available data were included. This led to slightly different sample sizes for the analyses of clinical data (AN = 36, HC = 42), metric BSE task (AN = 35, HC = 42), MEG data (AN = 29, HC = 30), and EEG data (AN = 32, HC = 34). Table 1 shows sample characteristics.

**Table 1:** Sample characteristics.

Group comparison	AN (*N* = 36)	HC (*N* = 42)	*t*(df)	*p*	*d*	
Age	15.33 ± 1.65	15.98 ± 1.81	*t*(76) = −1.62	*p* = 0.109	*d* = −0.369	n.s.
IQ	106.03 ± 13.18	106.64 ± 13.47	*t*(76) = −0.203	*p* = 0.840	*d* = −0.046	n.s.
BMI	15.60 ± 1.34	20.34 ± 1.98	*t*(76) = −12.10	*p* < 0.001	*d* = −2.75	AN < HC
BMI-SDS	−2.41 ± 1.00	−0.15 ± 0.64	*t*(58.15) = −11.55	*p* < 0.001	*d* = −2.71	AN < HC
EDE-I (ED interview)	3.47 ± 1.34	0.17 ± 0.19	*t*(36.25) = 14.58	*p* < 0.001	*d* = 3.57	AN > HC
EDI-C (ED questionnaire)	221.58 ± 54.60	98.95 ± 34.82	*t*(76) = 11.99	*p* < 0.001	*d* = 2.72	AN > HC
BSQ (Body Dissatisfaction)	119.00 ± 38.20	52.66 ± 16.48	*t*(46.29) = 9.65	*p* < 0.001	*d* = 2.30	AN > HC
BDI II (Depression)	26.28 ± 11.46	3.55 ± 4.65	*t*(44.81) = 11.14	*p* < 0.001	*d* = 2.67	AN > HC
SCARED (Anxiety)	29.63 ± 13.65	13.71 ± 7.66	*t*(51.26) = 6.13	*p* < 0.001	*d* = 1.47	AN > HC
*AN group clinical details*						
In-patient treatment duration in days		42.50 ± 65.50				
First in-patient treatment		*N* = 32	Repeated in-patient treatment			*N* = 4
Psychotropic medication		*N* = 7	Olanzapine			*N* = 3
			Escitalopram			*N* = 1
			Sertralin			*N* = 1
			Fluoxetin			*N* = 1
			Circadian			*N* = 1
Patients with comorbid disorders		*N* = 15	Depression			*N* = 10
			Specific phobia			*N* = 2
			Social anxiety disorder			*N* = 1
			OCD			*N* = 1
			PTBS			*N* = 1

*IQ* intelligence quotient, BMI body mass index, *BMI-SDS* body mass index standard deviation scores, *EDE-I* eating disorder examination interview, *EDE-C* eating disorder inventory for children, *BSQ* body shape questionnaire, *BDI II* Beck Depression Inventory 2, *SCARED* screen for child anxiety-related disorders.

### Behavioural data

#### Metric body size estimation task

**Fig. 2:**
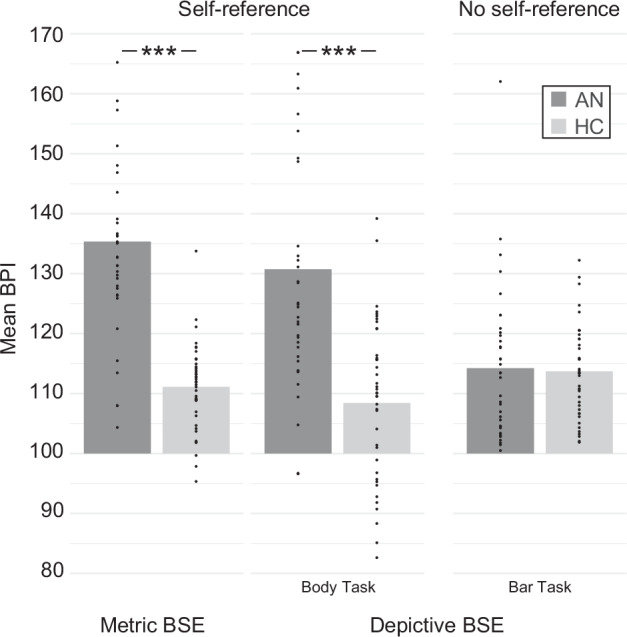
Body size estimation tasks. Compared to HC patients, AN patients showed higher BPIs and, thus, more overestimation in both BSE tasks involving self-reference (metric BSE task, body task) but similar BPIs in the BSE task without self-reference (bar task). AN in dark shades and HC in bright shades.

In the metric BSE task, AN patients had larger BPIs than HC, *t*(43,689) = 7.830, *p* < 0.001, *d* = 1.913 (Fig. 2, left).

#### Depictive body size estimation task

In the depictive BSE tasks, BPIs differed between AN and HC depending on the task, *task* × *group*: *F*(1,76) = 20.923, *p* < 0.001, *η*_p_^2^ = 0.216 (Fig. 2, right). In the body task, AN patients had larger BPIs than HC, *t*(58.71) = 5.36, *p* < 0.001, *d* = 1.25, but in the bar task, both groups had similar BPIs, *t*(56.03) = 0.215, *p* = 0.831, *d* = 0.051.

### Neural data

#### Group-independent effects

**Fig. 3:**
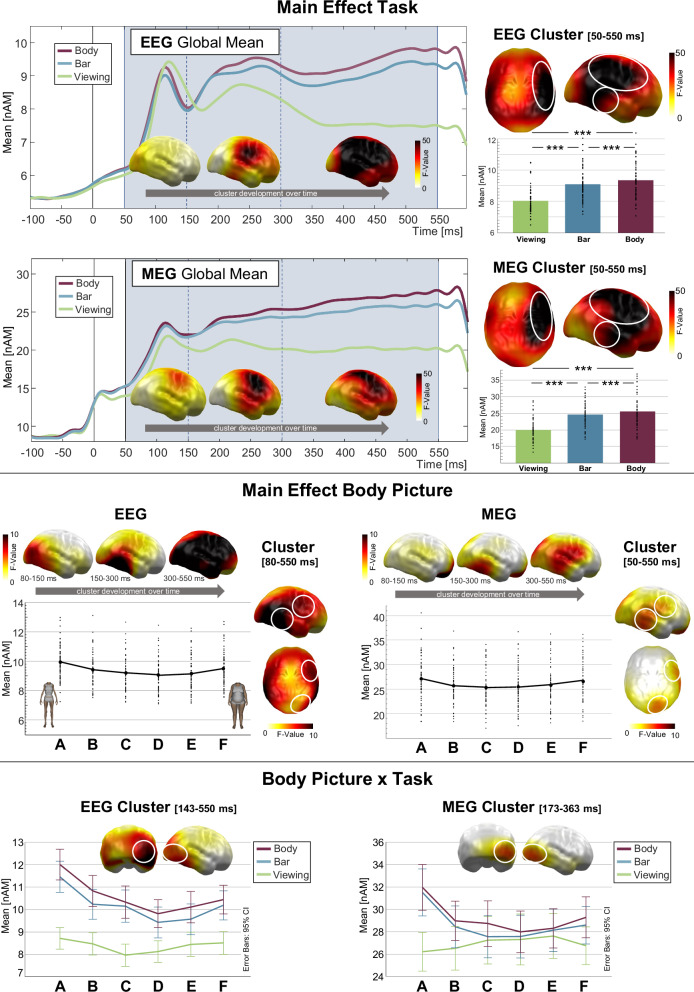
Group-independent effects. Top: Main effect of task. Top left: the global mean estimated neural source activity (nAm) from EEG and MEG data is plotted separately for the viewing, bar, and body tasks. The distribution of *F*-values within the respective cluster is projected onto a standard brain in three time windows (50–150, 150–300 and 300–550 ms). Top right: Distribution of *F*-values within the respective cluster averaged across the whole cluster duration (50–550 ms). White circles indicate regions with overlapping strong effects in both EEG and MEG. Bar graphs show post hoc comparisons of mean neural source activity in the viewing, bar and body tasks (***p = 0.001). The data of individual subjects are indicated by black dots (scatter plot). Middle: Main effect of body picture. The distribution of *F*-values within the respective EEG (left) and MEG (right) cluster is projected onto a standard brain in three time windows (50–150, 150–300 and 300–550 ms). Below, a line plot presents the mean neural activity in response to the six body picture categories (A–F). The data of individual subjects are indicated by black dots (scatter plot). To the right of the line plot the distribution of *F*-values within the respective cluster is shown averaged across the whole cluster duration (EEG: 80–550 ms; MEG: 50–550 ms). Bottom: Body picture × task interaction. A line plot presents the mean neural activity in response to the six body picture categories (A–F), separately for each task (viewing, bar, body). Error bars indicate a 95% confidence interval. On top of the line plot the distribution of *F*-values within the respective cluster is shown averaged across the whole cluster duration (EEG: 143–550 ms; MEG: 173–363 ms).

Both EEG and MEG analyses revealed significant clusters with a main effect of *task* across all analysed time points and across the brain. Estimated neural activity (in the following, “neural activity”) was stronger in the BSE tasks compared to the viewing task, and stronger in the body task compared to the bar task (Fig. 3). In EEG data, two adjacent clusters showing an identical main effect of *task* were merged into one cluster, *p*-cluster < 0.001, *F*(2,128) = 123.818, *p* < 0.001, *η*_p_^2^ = 0.659. Higher *F*-values indicating strong effects were first observed in the occipital cortex, then in a right lateralized fronto-parietal region and finally across the brain. Post hoc *t*-tests confirmed that all tasks differed (body vs. viewing: *t*(65) = 13.215, *p* < 0.001, *d* = 1.62; bar vs. viewing: *t*(65) = 11.224, *p* < 0.001, *d* = 1.71; body vs. bar: *t*(65) = 3.98, *p* < 0.001, *d* = 0.49). In MEG data, the main effect of *task* was also observed in an extended cluster, *p-*cluster < 0.001, *F*(2,114) = 97.374, *p* < 0.001, *η*_p_^2^ = 0.631. Strong effects occurred in a right dorsal fronto-parietal region early and then successively extended to the temporal cortex and became more bilateral. Post hoc *t*-tests again confirmed that all tasks differed (body vs. viewing: *t*(58) = 10.69, *p* < 0.001, *d* = 1.39; bar vs. viewing: *t*(58) = 11.55, *p* < 0.001, *d* = 1.50; body vs. bar: *t*(58) = 2.74, *p* = 0.008, *d* = 0.35). Overlapping regions showing a strong main effect of *task* in both the EEG and MEG were the right dorsal fronto-parietal and right temporal cortices.

Neural activity was particularly strong in response to pictures at BMI extremes, indicated by a main effect of *body picture* (Fig. 3). In EEG data, a respective cluster was observed from 80-550 ms across all neural sources, *p-*cluster < 0.001, *F*(5,320) = 52.232, *p* < 0.001, *η*_p_^2^ = 0.449. Strong effects started early in the bilateral occipital cortex, were then observed in the bilateral temporal cortex and finally nearly across the brain. Planned polynomial contrasts revealed a descending linear trend, *F*(1,64) = 39.982, *p* < 0.001, *η*_p_^2^ = 0.385, pointing towards greater activity for category A (lowest BMI), and a quadratic trend, *F*(1,64) = 160.714, *p* < 0.001, *η*_p_^2^ = 0.715. In MEG data, a respective cluster was observed across all time points and included the whole brain except the left DLPFC, *p-*cluster < .001, *F*(5,285) = 15.235, *p* < 0.001, *η*_p_^2^ = 0.211. Strong effects started early in a ventral frontal region, were then observed in the right temporal cortex and finally spread to the right fronto-parietal cortex. Planned polynomial contrasts revealed no linear trend, *F*(1,57) = 0.972, *p* = 0.328, *η*_p_^2^ = 0.017, but a strong quadratic trend, *F*(1,57) = 86.454, *p* < 0.001, *η*_p_^2^ = 0.603. Overlapping regions showing a strong main effect of *body picture* in both the EEG and MEG were the right ventral frontal and right temporal cortices.

Furthermore, the *body picture* effect differed between tasks. In EEG data, a *body picture* × *task* cluster occurred at 143–550 ms and was located at all but some orbitofrontal cortical areas, *p-*cluster < 0.001, *F*(10,640) = 9.661, *p* < 0.001, *η*_p_^2^ = 0.131. Strongest effects were located in the right occipito-temporal cortex. Planned polynomial contrasts indicated that tasks differed regarding *body picture* effects, with linear trends in the BSE tasks, body task, *F*(1,64) = 53.289, *p* < 0.001, *η*_p_^2^ = 0.454; bar task: *F*(1,64) = 36.476, *p* < 0.001, *η*_p_^2^ = 0.363, but not in the viewing task, *F*(1,64) = 0.569, *p* = 0.454, *η*_p_^2^ = 0.009. Quadratic trends occurred in all tasks, body task: *F*(1,64) = 85.744, *p* < 0.001, *η*_p_^2^ = 0.573; bar task: *F*(1,64) = 67.075, *p* < 0.001, *η*_p_^2^ = 0.512; viewing task: *F*(1,64) = 28.893, *p* < 0.001, *η*_p_^2^ = 0.311. In MEG data, a similar *body picture* × *task* cluster occurred at 173–363 ms and was located, overlapping with the strongest EEG effects, at the right occipito-temporal cortex, *p-*cluster = 0.015, *F*(10,570) = 10.024, *p* < 0.001, *η*_p_^2^ = 0.150. Planned polynomial contrasts again indicated that tasks differed regarding *body picture* effects, with linear trends in both BSE tasks, body task: *F*(1,57) = 11.355, *p* = 0.001, *η*_p_^2^ = 0.166; bar task: *F*(1,57) = 9.156, *p* = 0.004, *η*_p_^2^ = 0.138, but no linear trend in the viewing task, *F*(1,57) = 2.828, *p* = 0.098, *η*_p_^2^ = 0.047. Quadratic trends occurred in the BSE tasks, body task: *F*(1,57) = 34.375, *p* < 0.001, *η*_p_^2^ = 0.376; bar task: *F*(1,57) = 31.624, *p* < 0.001, *η*_p_^2^ = 0.357, but not in the viewing task, *F*(1,57) = 3.317, *p* = 0.074, *η*_p_^2^ = 0.055.

#### Group-specific effects

**Fig. 4:**
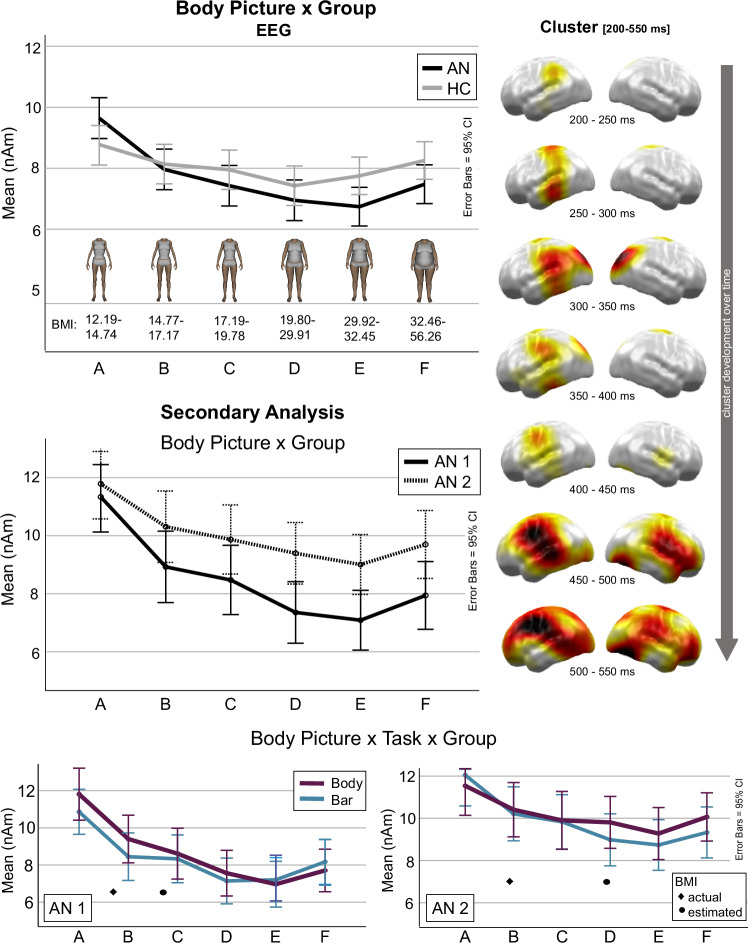
Group-specific EEG effects. Top: Body picture × group interaction. A line plot shows mean neural activity in response to the six body picture categories (A–F), separately for each group (AN in black and HC in clear grey lines). Body picture categories are shown with corresponding BMI range and example pictures. On the right side, the distribution of *F*-values within the respective cluster is projected onto a standard brain in seven time windows (200–550, 50 ms steps). Middle: Secondary analysis, body picture × group interaction comparing AN1 and AN2 subgroups. AN1 in solid black lines and AN2 in dashed black lines. Bottom: Secondary analysis, body picture × task × group interaction. Body task in red, bar task in blue lines. Mean actual (diamond) and estimated (dot) BMIs are shown for each subgroup (AN1, AN2).

In EEG data, the *body picture* effect differed between groups: A *body picture* × *group* cluster occurred at 200–550 ms and included all neural sources, *p-*cluster < 0.001, *F*(5,320) = 11.835, *p* < 0.001, *η*_p_^2^ = 0.480 (Fig. 4). The cluster started in the left parietal cortex, then extended to left temporal and shortly also to the bilateral occipital cortex, and finally involved bilateral fronto-parietal and occipito-temporal areas. Planned polynomial contrasts revealed group differences regarding both linear, *F*(1,64) = 24.952, *p* < 0.001, *η*_p_^2^ = 0.281, and quadratic trends, *F*(1,64) = 7.617, *p* = 0.008, *η*_p_^2^ = 0.106. Figure 4 shows a more descending linear trend in AN patients, with relatively higher responses to underweight (A) and relatively lower responses to higher weight (E, F) body pictures. No significant group-specific effects were observed in MEG data.

#### Secondary analysis in the EEG body picture × group cluster

The *body picture* effect also differed between AN1 (slight overestimation) and AN2 (distinct overestimation), *body picture* × *group*: *F*(3.735, 112.056) = 2.498, *p* = 0.033, *η*_p_^2^ = 0.077. Figure 4 shows a more descending linear trend in the AN1 compared to the AN2 group. Interestingly, the *body picture* effect in AN1 and AN2 also differed depending on the task, *body picture* × *task* × *group*: *F*(3.448, 103.445) = 3.311, *p* = 0.018, *η*_p_^2^ = 0.099. Planned polynomial contrasts revealed significant differences with respect to the linear trend, *F*(1,30) = 14.750, *p* < 0.001, *η*_p_^2^ = 0.330, but no differences regarding the quadratic trend, *F*(1,30) = 0.144, *p* = 0.707, *η*_p_^2^ = 0.005. Figure 4 shows that this is due to a more pronounced descending linear trend in the body (vs. bar) task in AN1 and an inverse effect in AN2.

Additional post hoc analyses in the EEG *body picture* × *group* cluster are reported in supplement S6.

## Discussion

This study used behavioural tasks and time-sensitive EEG and MEG to investigate the correlates of visual body perception and BSE in adolescent AN patients. As in prior studies [7], AN patients reported pronounced body dissatisfaction and overestimated their own body size more than controls in the self-referential BSE task. As body size overestimation also occurred in the metric BSE task, patients’ visual misjudgements in the depictive BSE task cannot likely be explained by methodological issues, e.g., the presented BMI range [53]. Nevertheless, AN patients did not misperceive body pictures in the non-self-referential depictive BSE task. Thus, our behavioural data suggest that BID in AN is more than uneasiness with one’s own body; it also includes the perception of one’s own body as wider than it is. However, our data do not suggest that these misjudgements are based on disruptions of visual body perception.

This view dovetails with the observation of normal neural source activity during early visual body processing in AN. Throughout the analysed time interval, AN patients and controls showed similar task- and stimulus-related effects (Fig. 3). The higher global neural activity in the BSE tasks compared to the viewing task is likely based on task relevance [54]. Higher activity in the body task compared to the bar task corresponds with the observation that self-reference enhances ERP amplitudes [55] and suggests that in the context of self-referential BSE, the emotional relevance of bodies is enhanced.

Regarding body picture BMIs, quadratic trends indicate stronger neural responses towards the more extreme (high and low) BMI categories, while a descending linear trend in the EEG suggests that this effect was (partly) more pronounced for body pictures at the low end of the BMI spectrum. This aligns with normative valence ratings showing a curvilinear relationship with picture BMIs: With increasingly extreme under- or overweight, ratings become more negative [34]. As emotional stimuli are known to capture attention and elicit enhanced neural processing in visual and fronto-parietal networks [56], this effect might be more pronounced for underweight body pictures because we included body pictures with more extreme low but less extreme high BMIs. Otherwise, this might reflect possibly ambivalent or positive evaluations of underweight body pictures. Two prior studies revealed more positive ratings and enhanced activity in the ventral striatum, associated with reward, in response to underweight body stimuli in adult AN patients [20, 21]. Underweight body stimuli were also rated as more positive by adolescent AN patients, but differences to controls were smaller [32]. Thus, healthy adolescent girls might also consider underweight bodies as positive. As stimuli with both positive and negative hedonic valence elicit similar emotional attention effects [57], both might contribute to the observed effects. In mid-latency to late time intervals, and in the occipital and the right temporal cortex in MEG and globally in EEG data, the emotional attention effect was presumably intensified by task-related attention processes [58]: Descending linear trends, and also quadratic trends in MEG data, were evident particularly in the BSE tasks (vs. viewing). Overall, across groups and across EEG and MEG data, results revealed plausible general and task-related emotional attention effects that were most pronounced in right lateralized fronto-parietal and temporal cortical regions, suggesting that our task design worked well.

Against this background, it is interesting to see a mid- to late latency group-specific effect in EEG data, with stronger linear and quadratic trends and (numerically) relatively higher neural activity in response to underweight and relatively lower neural activity in response to higher-weight body pictures in AN vs. HC (Fig. 4, top). This corresponds with previous reports of decreasing ERP amplitudes from underweight to normal weight to overweight body stimuli in AN [31, 32]. It presumably reflects more pronounced positive [20, 21] and/or negative [34] emotions in response to underweight body stimuli in AN, which leads to a greater capture of attention and deeper processing. In our view, the fact that this effect started mid-latency and involved enhanced (not reduced) neural activity in widespread brain regions argues against a disturbance specific to the neural network of visual body processing in AN but in favour of a disturbance in the cognitive-affective domain.

Nevertheless, we did not find the expected manifestation of cognitive-affective processes, particularly in the body task. In this regard, although it is certainly underpowered, our secondary analysis revealed a glimpse of possible neural correlates of self-referential (vs. non-self-referential) BSE (Fig. 4): AN patients showing slight overestimation (AN1) seemed to respond particularly strongly to body pictures with very low BMI, near their actual/estimated BMI, while AN patients showing strong overestimation (AN2) responded relatively more to body pictures with normal to high BMI, near their estimated (not actual) BMI. This might reflect the allocation of attention toward body pictures with which AN patients are identified. It suggests that the neural correlates of BSE depend on both the individual actual BMI and the extent of overestimation and, thus, require more individualised, differentiated data analyses. Unfortunately, such methods require bigger sample sizes to provide sufficient statistical power.

Further limitations are, first, that the study included adolescent restrictive-type AN patients during treatment. Thus, conclusions cannot necessarily be generalised to adult patients, patients with binge-purge-type AN, or untreated patients. However, our focus on a relatively homogenous sample strengthens the informative value for this specific group. Second, while many prior studies used individual photographs, we employed highly standardised computer-generated body pictures, probably leading to a comparatively lower ecological validity. Nonetheless, the paradigm was designed to present many stimuli in a short time and keep all stimuli identical between subjects, allowing for a good signal-to-noise ratio and high comparability. Third, some newer BSE tasks take better account of individual body shapes and might thus have revealed different results [53]. Fourth, due to data loss, our analyses were based on slightly different samples, which might partly account for discrepancies between different data sources, e.g. EEG vs. MEG results. Finally, we did not obtain valence ratings, so respective interpretations were based on other data [34]. This is a clear limitation, as we cannot be sure about the subjective evaluation of the body pictures of our subjects or their idea of an ideal body. However, body picture valence was not the focus of the study, and respective ratings were omitted due to time constraints. Still, future studies should ask participants to rate picture valence.

Overall, our study revealed that BID in adolescent restrictive-type AN patients likely depends on self-reference. Intact estimations in a non-self-referential BSE task and normal early neural visual processing of body stimuli speak against a disturbance of visual perception. Instead, enhanced mid- to late latency and regionally distributed neural activity in response to self-relevant body stimuli in AN suggest a central role of biased emotional attention. With regard to the treatment of body image disturbance in AN, this argues against a focus on the correction of visual misperception and in favour of a focus on emotional body experience.

## Supplementary information

Supplementary Material

Table S4: Correlations of BMI-SDS, BSQ, and BPI in the Body Task with neural activity in the EEG body task x group cluster.

## Footnotes

1 EEG and MEG compensate for several of each other’s technical shortcomings, and thus a combination of both methods provides more accurate, complementary localisation results [33].

2 Neurologic or other somatic disorders with known influence on the central nervous system, defects of vision or hearing that cannot be sufficiently corrected, metal or metalliferous objects in or at the body

3 Note that source estimations of EEG data were based on 57 or 80 EEG channels, respectively, but both resulted in 350 dipole triplets. Because in a spherical model radial sources do not generate measurable magnetic fields outside the head, dipole pairs (azimuthal and polar directions only) were estimated for MEG, and dipole triplets (azimuthal, polar, and radial directions) were estimated for EEG.

4 Note that the ANOVA used positive and negative BPI values, representing over- and underestimation, for the body task but only positive BPI values, representing the general estimation error, for the bar task. ANOVA results did not change substantially when positive and negative values were also used in the bar task.
