# Supplementary material for: Visual body size estimation in adolescent anorexia nervosa: Behavioural and neurophysiological data suggest intact visual perception and biased emotional attention
Source: Transl Psychiatry. 2024 Oct 18;14:442. doi: 10.1038/s41398-024-03144-y (PMC11489811; doi:10.1038/s41398-024-03144-y)
Supplement: Supplementary file 1 — Supplementary Material [file 41398_2024_3144_MOESM1_ESM.docx]

**Supplementary Material**

**S1: EEG electrode configuration**

About halfway through the data collection interval, the electronics of the MEG system were replaced. With the old electronics, 80 EEG channels could be recorded in parallel to the MEG. After the upgrade, the maximum number of EEG channels to be recorded in parallel was reduced to 64, of which 57 channels were then used. The scalp-coverage of the 57 and 80 electrode positions was identical, but the electrode density was reduced accordingly.

**S1A: EEG electrode configuration with 80 channels**

This EEG electrode configuration with 80 channels was used at the beginning of the study period in N = 19 HC participants and N = 20 AN Patients**.**


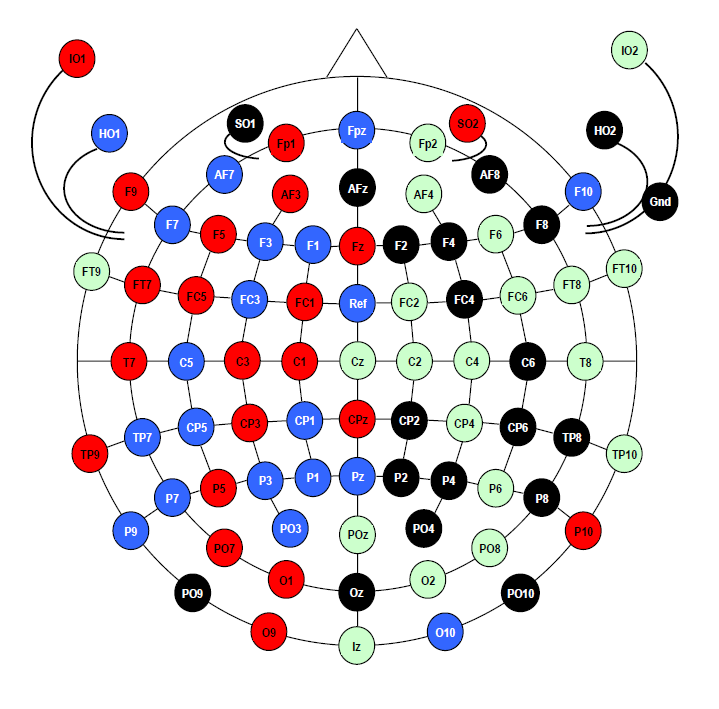


**S1B: EEG electrode configuration with 57 channels**

This EEG electrode configuration with 57 channels was used after the technical changes in N = 27 HC participants and N = 18 AN patients**.**


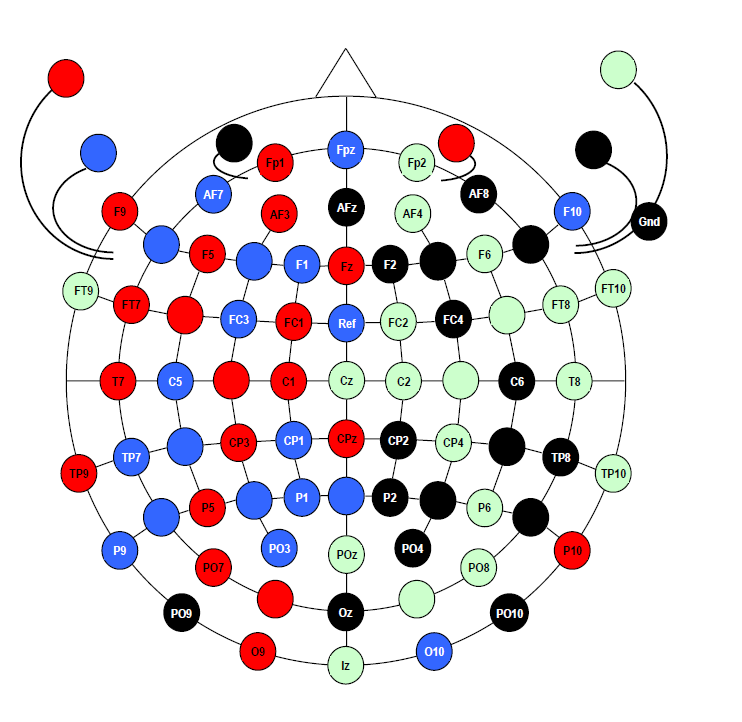


**S2 Overview of EEG electrode data and MEG sensor data**

**
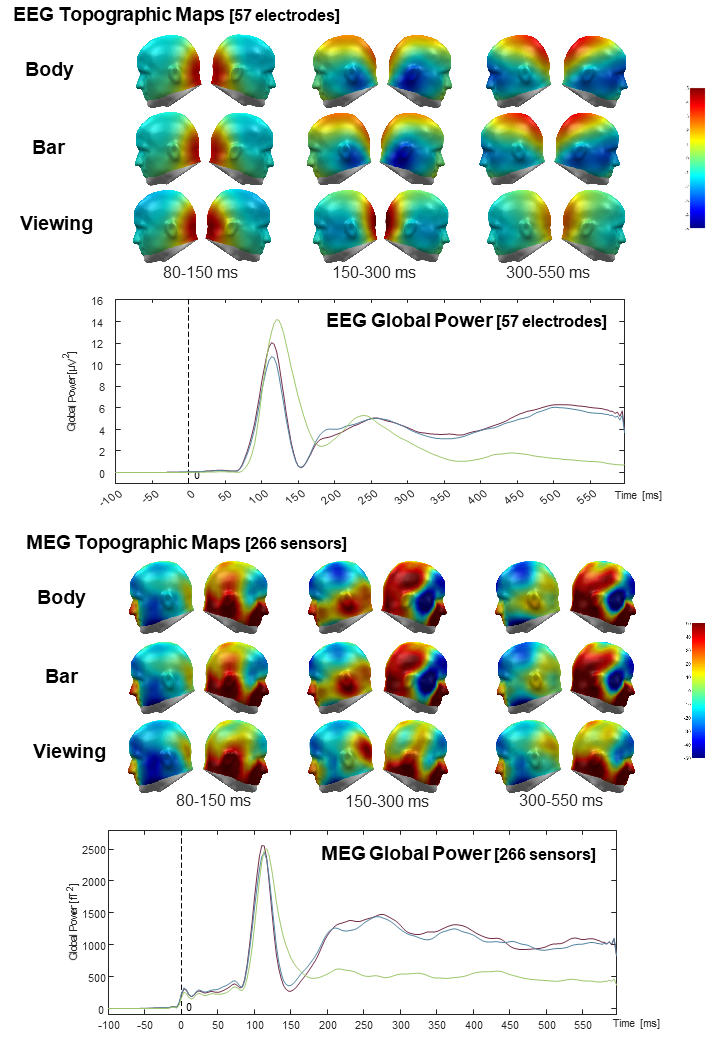
**

**S3 Sample Characteristics of AN subgroups AN1 and AN2**

|  | AN1 [*N*=16] | AN2 [*N*=16] | *t* | *df* | *p* | *d* |  |
| --- | --- | --- | --- | --- | --- | --- | --- |
| Age | 15.63±1.50 | 14.75±1.80 | 1.49 | 30 | .147 | 0.527 | n.s. |
| IQ | 105.50±15.49 | 107.00±11.77 | -0.30 | 30 | .760 | -0.109 | n.s. |
| BMI | 15.03±0.86 | 16.19±1.56 | -2.60 | 23.40 | .016 | -0.897 | AN1<AN2 |
| BMI-SDS | -2.85±0.77 | -1.89±1.03 | -2.97 | 30 | .006 | -1.05 | AN1<AN2 |
| EDE-I | 3.14±1.51 | 3.73±1.26 | -1.18 | 30 | .245 | -0.419 | n.s. |
| EDI-C | 202.38±60.76 | 239.56±49.03 | -1.90 | 30 | .066 | -0.674 | n.s. |
| BSQ | 59.27±10.71 | 63.87±9.99 | -1.21 | 28 | .234 | -0.444 | n.s. |
| BDI II | 21.25±12.09 | 31.25±9.60 | -2.59 | 30 | .015 | -0.916 | AN1<AN2 |
| SCARED | 26.67±15.14 | 33.19±13.15 | -1.28 | 29 | .210 | -0.461 | n.s. |
| Bar task  estim. BMI | 20.35±4.37 | 20.02±3.12 | 0.25 | 30 | .804 | -0.089 | n.s. |
| Body task  estim. BMI | 17.42±1.52 | 23.92±4.23 | -5.78 | 18.81 | <.001 | -1.99 | AN1<AN2 |

|  | AN1 [*N*=16] | HC [*N*=34] | *t* | *df* | *p* | *d* |  |
| --- | --- | --- | --- | --- | --- | --- | --- |
| Age | 15.63±1.50 | 15.91±1.91 | -0.527 | 48 | .600 | -0.160 | n.s. |
| IQ | 105.50±15.49 | 107.47±14.38 | -0.441 | 48 | .661 | -0.134 | n.s. |
| BMI | 15.03±0.86 | 20.47±2.04 | -10.18 | 48 | <.001 | -3.08 | AN1<HC |
| BMI-SDS | -2.85±0.77 | -0.08±0.62 | -13.49 | 48 | <.001 | -4.09 | AN1<HC |
| EDE-I | 3.14±1.51 | 0.20±0.20 | 7.72 | 15.25 | <.001 | 3.34 | AN1>HC |
| EDI-C | 202.38±60.76 | 97.74±32.49 | 6.46 | 19.15 | <.001 | 2.37 | AN1>HC |
| BSQ | 59.27±10.71 | 35.38±9.46 | 5.59 | 17.91 | <.001 | 1.70 | AN1>HC |
| BDI II | 21.25±12.09 | 3.68±4.98 | 5.59 | 17.44 | <.001 | 2.18 | AN1>HC |
| SCARED | 26.67±15.14 | 13.76±7.66 | 3.12 | 17.25 | .006 | 1.21 | AN1>HC |
| Bar task  estim. BMI | 20.35±4.37 | 21.33±4.23 | -0.757 | 48 | .453 | -0.229 | n.s. |
| Body task  estim. BMI | 17.42±1.52 | 22.27±2.58 | -6.93 | 45.38 | <.001 | -2.07 | AN1<HC |
|  |  |  |  |  |  |  |  |

|  | AN2 [*N*=16] | HC [*N*=34] | *t* | *df* | *p* | *d* |  |
| --- | --- | --- | --- | --- | --- | --- | --- |
| Age | 14.75±1.80 | 15.91±1.91 | -2.03 | 48 | p=.047 | d=-0.608 | AN2<HC |
| IQ | 107.00±11.77 | 107.47±14.38 | -.114 | 48 | p=.910 | d=.035 | n.s. |
| BMI | 16.19±1.56 | 20.47±2.04 | -7.40 | 48 | p<.001 | d=-2.24 | AN2<HC |
| BMI-SDS | -1.89±1.03 | -0.08±0.62 | -6.43 | 20.34 | p<.001 | d=-2.28 | AN2<HC |
| EDE-I | 3.73±1.26 | 0.20±0.20 | 11.10 | 15.36 | p<.001 | d=4.78 | AN2>HC |
| EDI-C | 239.56±49.03 | 97.74±32.49 | 12.17 | 48 | p<.001 | d=3.69 | AN2>HC |
| BSQ | 63.87±9.99 | 35.38±9.46 | 10.29 | 19.29 | p<.001 | d=3.76 | AN2>HC |
| BDI II | 31.25±9.60 | 3.68±4.98 | 10.82 | 18.91 | p<.001 | d=4.00 | AN2>HC |
| SCARED | 33.19±13.15 | 13.76±7.66 | 6.59 | 48 | p<.001 | d=1.99 | AN2>HC |
| Bar task  estim. BMI | 20.02±3.12 | 21.33±4.23 | 0.25 | 48 | p=.273 | d=-0.336 | n.s. |
| Body task  estim. BMI | 23.92±4.23 | 22.27±2.58 | -5.78 | 20.44 | p=.167 | d=0.507 | n.s. |

**S4 Determination of the required sample size**

The required sample size for this study was determined using G*Power 3 (Faul et al., 2007) based on effect sizes from Horndasch et al. (2018). This study was selected because - compared to all other EEG/MEG studies on body image in Anorexia nervosa that were available at the time - the study design was considered as most comparable to the present study. In this study, pictures of bodies in five weight categories (extremely underweight, underweight, normal weight, overweight, extremely overweight) were presented to AN patients and HC participants in a passive viewing paradigm. For a significant interaction of ‘category`’ and ‘group’ (AN, HC) Horndasch and colleagues reported *η_p_^2^*=0.07, which corresponds to cohens f = 0.27. For an effect size of f = 0.27, an alpha-level of 0.05, a power (1-ß) of 0.95, 2 groups and 6 catergories (A to F), G*Power 3 revealed a total sample size of N = 24.

As we were specifically interested in early effects (corresponding to the P100 time interval), we also considered the studies of Li et al. (2015, 2016) during the planning of the required sample size. In these studies, face and house stimuli were presented to AN patients and HC participants in a visual matching task and the authors reported a general lower response to these stimuli in AN vs. HC in early P100 time intervals. Unfortunately, Li and colleagues did not report any effect sizes. Nevertheless, as the respective significant effects were reported in samples of N = 15 or N = 20 per group, respectively, we were confident that the planned sample size of at least N = 24 participants per group will be sufficient to discover comparable effects in the present study.

**S5: Flow Chart of the in- and exclusion process**

**
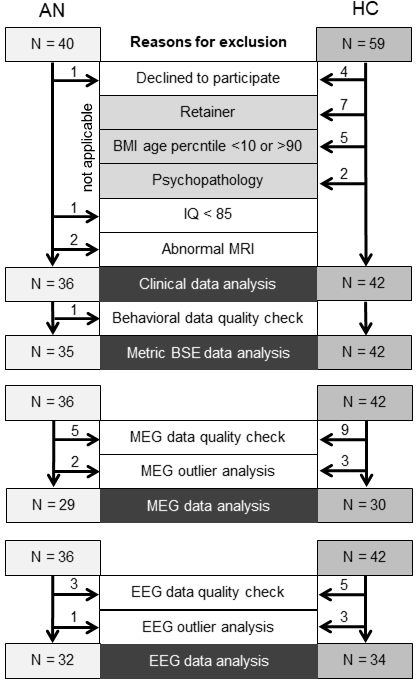
**

**S6 Additional post hoc analyses in the EEG *Body-Picture x Group* cluster**

Note that these additional statistics are uncorrected for multiple comparisons.

**Polynomial contrasts for the factor Body-Picture in AN and HC separately:**

AN patients

Body-Picture linear trend *F*(1,31)=72.443, *p*<.001, *η_p_^2^*=0.700

Body Picture quadratic trend *F*(1,31)=118,263, *p*<.001, *η_p_^2^*=0.792

HC participants

Body-Picture linear trend *F*(1,31)=11.866, *p*=.002, *η_p_^2^*=0.264

Body-Picture quadratic trend *F*(1,31)=23.216, *p*<.001, *η_p_^2^*=0.413

**Post hoc t-tests comparing AN and HC in each Body-Picture category:**

**A:** *T*(64)=1.855, *p*=.068, *d*=1.90; AN: M=10.18, SD=1.73; HC: M=9.31, SD=2.04

**B:** *T*(64)=-0.374, *p*=.709, *d*=1.89; AN: M=8.50, SD=1.82; HC: M=8.67, SD=1.96

**C:** *T*(64)=-1.128, *p*=.263, *d*=1.88; AN: M=7.96, SD=1.71; HC: M=8.48, SD=2.03

**D:** *T*(64)=-1.029, *p*=.0.307, *d*=1.89; AN: M=7.48, SD=1.79; HC: M=7.96, SD=1.98

**E:** *T*(64)=-2.291, *p*=.025, *d*=1.79; AN: M=7.27, SD=1.68; HC: M=8.28, SD=1.90

**F:** *T*(64)=-1.753, *p*=.084, *d*=1.80; AN: M=8.01, SD=1.89; HC: M=8.79, SD=1.71

**Polynomial contrasts for the factor Body-Picture (across Body task and Bar task) in AN1 and AN2 separately:**

AN1 (slight overestimation)

Body-Picture linear trend *F*(1,15)=119.197, *p*<.001, *η_p_^2^*=0.888

Body Picture quadratic trend *F*(1,15)=53.547, *p*<.001, *η_p_^2^*=0.781

AN2 (distinct overestimation)

Body-Picture linear trend *F*(1,15)=16.757, *p*<.001, *η_p_^2^*=0.528

Body Picture quadratic trend *F*(1,15)=26.895, *p*<.001, *η_p_^2^*=0.642

**Post hoc t-tests comparing AN1 and AN2 in each Body-Picture category (across Body task and Bar task):**

**A:** *T*(30)=-0.539, *p*=.594, *d*=2.37; AN1: M=11.34, SD=2.15; AN2: M=11.79, SD=2.57

**B:** *T*(30)=-1.626, *p*=.114, *d*=2.41; AN1 M=8.92, SD=2.03; AN2: M=10.31, SD=2.74

**C:** *T*(30)=-1.687, *p*=.102, *d*=2.34; AN1: M=8.47, SD=2.03; AN2: M=9.87, SD=2.47

**D:** *T*(30)=-2.780, *p*=.009, *d*=2.07; AN1: M=7.35, SD=1.90; AN2: M=9.39, SD=2.23

**E:** *T*(30)=-2.689, *p*=.012, *d*=2.02; AN1: M=7.09, SD=1.97; AN2: M=9.01, SD=2.21

**F:** *T*(30)=-2.170, *p*=.038, *d*=2.29; AN1: M=7.94, SD=1.97; AN2: M=9.70, SD=2.57

**Polynomial contrasts for the factor Body-Picture for each Task (Viewing task, Body task, Bar task), each AN subgroup (AN1, AN2), and the HC group separately:**

**Viewing task**

AN1 (slight overestimation)

Body-Picture linear trend *F*(1,15)=5.367, *p*=.035; *η_p_^2^*=0.264

Body-Picture quadratic trend: *F*(1,15)=30.565, *p*<.001; *η_p_^2^*=0.67

AN2 (distinct overestimation)

Body-Picture linear trend: *F*(1,15)=7.290, *p*=.016; *η_p_^2^*=0.327

Body-Picture quadratic trend: *F*(1,15)=51.224, *p*<.001; *η_p_^2^*=0.773

HC participants

Body-Picture linear trend: *F*(1,33)=7.718, *p*=.009; *η_p_^2^*=0.190

Body-Picture quadratic trend: *F*(1,33)=19.173, *p*<.001; *η_p_^2^*=0.367

**Bar task**

AN1 (slight overestimation)

Body-Picture linear trend: *F*(1,64)=32.339, *p*<.001; *η_p_^2^*=0.683

Body-Picture quadratic trend: *F*(1,64)=38.530, *p*<.001; *η_p_^2^*=0.720

AN2 (distinct overestimation)

Body-Picture linear trend: *F*(1,64)=57.980, *p*<.001; *η_p_^2^*=0.794

Body-Picture quadratic trend: *F*(1,64)=23.979, *p*<.001; *η_p_^2^*=0.615

HC participants

Body-Picture linear trend: *F*(1,64)=1.927, *p*=.174; η_p_^2^=0.055

Body-Picture quadratic trend: *F*(1,64)=17.196, *p*<.001; η_p_^2^=0.342

**Body task**

AN1 (slight overestimation)

Body-Picture linear trend: *F*(1,64)=176.228, *p*<.001; η_p_^2^=0.922

Body-Picture quadratic trend: *F*(1,64)=33.485, *p*<.001; η_p_^2^=0.691

AN2 (distinct overestimation)

Body-Picture linear trend: *F*(1,64)=4.530, *p*=.050; η_p_^2^=0.232

Body-Picture quadratic trend: *F*(1,64)=7.510, *p*=.015; η_p_^2^=0.334

HC participants

Body-Picture linear trend: *F*(1,64)=10.447, *p*=.003; η_p_^2^=0.240

Body-Picture quadratic trend: *F*(1,64)=11.036, *p*=.002; η_p_^2^=0.251

**Post hoc correlation analyses:**

Results are presented in Table S5A (separate file)

Note that the post hoc correlation analyses are not corrected for multiple comparisons and have to be interpreted with caution.

Post hoc correlation analyses revealed that in AN patients more overestimation of their own body size during self-referential depictive BSE (BPI Body Task) was associated with higher relative weight (BMI-SDS) and higher body dissatisfaction (BSQ). This might be related to the fact that AN patients with very low body weight showed less body size overstimation (and possibly less body dissatisfaction, as might be hinted by the near trend level correlation of BMI-SDS with BSQ above). This fits with the results reported in S1: Group AN1 with slight overestimation has a lower mean relative weight (BMI, BMI-SDS) compared to group AN2 with distinct overestimation In HC participants, on the contrary, estimation of a relatively bigger than actual body size (BPI Body Task) was associated with lower relative weight (BMI-SDS), but not with body dissatisfaction. This might reflect that HC participants with higher relative weight tend to correct estimations or even underestimations of their body size (note that a numer of HC participants underestimated their body size, as can be seen in Figure 2). This seems to be unrelated to body dissatisfaction.

Interestingly, AN patients also showed negative correlations of their relative weight (BMI-SDS) and body size overestimation (BPI Body Task) with a relative higher neural response to underweight body pictures (category A) in the bar compared to the body task (Cluster Mean Category A Body – Bar). This again fits with the results reported in the comparision of the AN1 vs. AN2 groups (Figure 4). Especially AN patients with both a lower relative weight (BMI-SDS) and lower body size overestimation (BPI body task) show stronger responses to underweight body pictures – best reflecting their actual body size – during the body task.

Likewise, in line with the results of our secondary analyses comparing AN1 vs AN2, more body size overestimation (BPI Body Task) in AN patients was related with stronger neural responses to overweight body pictures (Cluster Mean Category F Body + Body minus Bar (trend)) – presumably reflecting greater caption of attention by stimuli near the estimated own body size. In this case, there was not a correlation with the relative weight (BMI-SDS), but with body dissatisfaction. In short: AN patients with more body dissatisfaction responded more to overweight body pictures. A similar correlation occurred in the HC group: HC participants with more body dissatisfaction showed relatively stronger responses to overweight body pictures in the body versus bar task. However, this seems to be, at least in part, driven by a negative correlation of body dissatisfaction with neural responses to overweight body pictures in the bar task.
