# Supplementary material for: Visual body size estimation in adolescent anorexia nervosa: Behavioural and neurophysiological data suggest intact visual perception and biased emotional attention
Source: Transl Psychiatry. 2024 Oct 18;14:442. doi: 10.1038/s41398-024-03144-y (PMC11489811; doi:10.1038/s41398-024-03144-y)
Supplement: Supplementary file 2 — Table S4: Correlations of BMI-SDS, BSQ, and BPI in the Body Task with neural activity in the EEG body task x group cluster. [file 41398_2024_3144_MOESM2_ESM.docx]

**Table S4: Correlations of BMI-SDS, BSQ, and BPI in the Body Task with neural activity in the EEG *body task x group* cluster.**

| Group | | | | | | BMI-SDS | BSQ | BPI Body Task | Cluster Mean Global (nAm) | Cluster Mean Category A  Bar Task | Cluster Mean Category A Body Task | Cluster Mean Category A Body - Bar | Cluster Mean Category F  Bar Task | Cluster Mean Category F Body Task | Cluster Mean Category F Body - Bar |
| --- | --- | --- | --- | --- | --- | --- | --- | --- | --- | --- | --- | --- | --- | --- | --- |
| AN | BMI-SDS | | Pearson correlation | | | 1 | .275 | **.462**** | .231 | .176 | -.314 | **-.605**** | .212 | .287 | .104 |
|  |  |  | Sig. (2-sided) | | |  | .105 | .005 | .204 | .336 | .080 | <.001 | .244 | .111 | .570 |
|  |  |  | N | | | 36 | 36 | 36 | 32 | 32 | 32 | 32 | 32 | 32 | 32 |
|  | BSQ | | Pearson correlation | | | .275 | 1 | **.507**** | .259 | .131 | -.055 | -.238 | .142 | **.413*** | **.376*** |
|  |  |  | Sig. (2-sided) | | | .105 |  | .002 | .152 | .475 | .766 | .189 | .438 | .019 | .034 |
|  |  |  | N | | | 36 | 36 | 36 | 32 | 32 | 32 | 32 | 32 | 32 | 32 |
|  | BPI Body Task | | Pearson correlation | | | .462** | .507** | 1 | .351* | .166 | -.281 | **-.553**** | .211 | **.454**** | .338 |
|  |  |  | Sig. (2-sided) | | | .005 | .002 |  | .049 | .364 | .120 | .001 | .247 | .009 | .059 |
|  |  |  | N | | | 36 | 36 | 36 | 32 | 32 | 32 | 32 | 32 | 32 | 32 |
| HC | BMI-SDS | | Pearson correlation | | | 1 | .299 | **-.340*** | -.199 | -.033 | -.142 | -.159 | -.251 | -.155 | .126 |
|  |  |  | Sig. (2-sided) | | |  | .058 | .028 | .260 | .851 | .422 | .371 | .152 | .382 | .477 |
|  |  |  | N | | | 42 | 41 | 42 | 34 | 34 | 34 | 34 | 34 | 34 | 34 |
|  | BSQ | | Pearson correlation | | | .299 | 1 | .081 | -.234 | -.222 | -.055 | .137 | **-.400*** | .009 | **.522**** |
|  |  |  | Sig. (2-sided) | | | .058 |  | .615 | .190 | .213 | .761 | .448 | .021 | .961 | .002 |
|  |  |  | N | | | 41 | 41 | 41 | 33 | 33 | 33 | 33 | 33 | 33 | 33 |
|  | BPI Body Task | | Pearson correlation | | | -.340* | .081 | 1 | .149 | -.025 | .037 | .073 | .179 | .242 | .077 |
|  |  |  | Sig. (2-sided) | | | .028 | .615 |  | .402 | .889 | .836 | .682 | .311 | .169 | .666 |
|  |  |  | N | | | 42 | 41 | 42 | 34 | 34 | 34 | 34 | 34 | 34 | 34 |
|  | |  | |  | **p<0.01. *p<0.05 | | | | | | | | | | |
